# Mr. Cooke's Abridgment of Morgagni on the Seats and Causes of Diseases

**Published:** 1822-12-01

**Authors:** 


*The Seats and Causes of Diseases, investigated by Anatomy; containing a great Variety of Dissections, and accompanied with Remarks.* By John Baptist Morgagni, Chief Professor of Anatomy, and President of the University at Padua. Abridged and elucidated with copious Notes, by William Cooke, Member of the Royal College of Surgeons, London; and one of the Secretaries to the Hunterian Society. In two volumes, 8vo, pp. 577 and 693. London, 1822.

The original work of Morgagni has been oftener quoted than read—and this translation of it will be oftener read than quoted. It is very easy to account for this. Those who *write*, and have occasion to refer to Morgagni's collection, will prefer the original, were it only for the look of the thing; while those who *read*, for the sake of storing their minds with useful facts, or assisting their judgment in *private* practice, will take the shorter route of a condensed translation, as sufficiently accurate and comprehensive for their purposes. There were several circumstances that rendered the original work, and even Alexander's translation of it almost unknown (except by name) to the great body of the profession. The size alone was an insuperable obstacle. Not one medical man now in a thousand has nerve to encounter *three huge quartos*, on any subject, or in any language. The taste for research, too, among the records of the past, is daily declining—even among those who have time for such explorations. The multiplicity of modern productions renders it extremely difficult for the practitioner to keep pace with the ever toiling press—and to rake up the rubbish of antiquated lore for the chance of finding a few valuables, is quite out of the question. Indeed, when these selectæ are brought forward by a few learned and industrious men, they are too often neglected, and the collector laughed at for his pains. No wonder then, that, under such encouragement from the public, authors should now-a-days content themselves with the relation of single histories, or the statement of insulated facts, hardly venturing even to draw a conclusion, lest they should be stigmatized as theorists.

In respect to Morgagni's work, it is an immense magazine of facts, (some true and perhaps many false,) diluted, in most instances, by unnecessary verbiage, and disfigured, in many places, by antiquated doctrines, both of pathology and physiology. A separation therefore of the facts from the extraneous matters surrounding them, is desirable in itself, and likely to suit the taste of the times. In the second volume of the Medico-Chirurgical Journal, for the concluding six months of the year 1816, we *translated* and *condensed* the first five epistles of Morgagni upon the same plan precisely as the present work is constructed;* and to the then limited number of our subscribers the project gave very general satisfaction. Circumstances, which it is unnecessary to state, occurred, which arrested our progress, and we are glad to see our plan acted on and completed by Mr. Cooke, though he has not made any allusion to our priority in the field.

Viewing the original work, then, as a collection of facts or cases, we consider the arrangement as of very subordinate consequence. Mr. Cooke has deviated from the order of the original; but whether with advantage or not, we really cannot say, and do not think it necessary to enquire. We are glad to observe that Mr. Cooke has corrected several errors into which Dr. Alexander had fallen—an example of which occurs in one of the first cases translated by the latter gentleman, viz. cardinal sanvitalis, where Dr. A. has put convulsive motions of the hands and *feet*, for convulsive motions of the hands and *face*:—"convulsivis motibus in *facie* et manibus."

In respect to the propriety of appending notes, we are rather disposed to think that they would have been better omitted. We acknowledge that we proceeded, as far as we went, on the same plan as Mr. Cooke; but were we to go over the same ground again, we should prefer condensing the whole into one volume by means of small type, to that of swelling out the work into two volumes by open spaces, large type, and notes. The circulation of the work would thus have been increased five-fold. By this observation we do not mean to undervalue the appended matter, but only to query the policy of the measure. It will be for Mr. Cooke to take this into consideration in a subsequent edition, which we have no doubt will soon be called for, notwithstanding the disinclination of readers in general, of the present day, to try back into the archieves of former times.

A publication of this description bids defiance to regular analysis, being itself, in fact, an analytical digest of a very voluminous production. We shall, however, select such a group of cases from certain divisions of the work as may prove, at once, interesting in themselves, and indicative of the nature and execution of the whole performance.

---

* See Vol. II. pp. 159—254—430—512.

Before entering on our labours, we may be permitted to state a few biographical particulars respecting the illustrious dead. Morgagni was born at Forli in Italy, in February 1682, and studied medicine with great ardour at Bologna, under Valsalva and Albertini. He afterwards visited Venice and Padua. In the latter university he became chief anatomical professor in the year 1715, and thenceforth, till the close of a long life, deservedly ranked at the head of the anotomists of his time. Literary honours were, of course, accumulated on his head, and he was visited by all the literati who came into his neighbourhood. He married a lady of noble family, and had fifteen children, eight of whom survived him. He was of a robust habit, tall, and of a lively and agreeable countenance. His other virtues were also enhanced by his great modesty. He died about the end of 1771, at the advanced age of ninety, but in the possession of his mental faculties. He was the friend and cotemporary of the celebrated Lancisi, Vallisneri, and Valsalva. His great work, the "Adversaria Anatomica," was published in parts, between the years 1706 and 1719, and extended its author's fame all over Europe. The present work, "De Sedibus et Causis Morborum," did not appear till the year 1760, when the author had nearly attained his 80th year. In 1769 Dr. Alexander published a translation in three quarto volumes, which, from their bulkiness chiefly, have been confined to the libraries of a very few, comparatively speaking. The circulation of the original was also very confined. The great fault (and a most important one it is) in Morgagni's work is the defective history of each case. In the majority of instances there is nothing that can be called a history of the disease prior to death, and in those that are more detailed the symptomatology is extremely imperfect, and the effects of remedies, or modes of treatment, are but seldom adverted to. Yet, after every deduction, we are disposed to agree in sentiment with our own illustrious pathologist and physician, Dr. Baillie, that "when considered in all its parts it would be difficult to bestow upon it too high praise." But we must now proceed to the more direct objects of our labours.

The first five or six sections of the work will not detain us long, since they are on diseases of the membranes and substance of the brain, particularly apoplexy—affections which have lately occupied much of this journal, and which have been investigated by modern pathologists in a manner far more accurate and satisfactory than Morgagni could lay claim to. We shall here, however, condense a case from Valsalva, which is not uninteresting.

"A woman, 70 years of age, had for many months felt a gradual decay of memory, and also of vision, when objects were placed in a certain position. When walking, she scarcely raised her feet from the ground. She had been seized, about twelve months previously, with a disorder in the head, from which she speedily recovered; but now she fell down suddenly, while eating, with hemiplegia of the left side, and paralysis of the right arm. Her respiration was perfectly natural, as was also the colour of her face, which, in her, was pale; nor did any convulsions appear. Her head fell, like that of a corpse, into any position to which it was thrown. She exhibited no sense of understanding or feeling, except a slight degree of motion when an incision was made into the jugular vein. She died in nine hours. Both ventricles of the brain were filled with fluid blood; the right, much eroded, as well about the external margin of the corpus striatum as of the thalamus nervi optici. The plexus choroides could scarcely be observed. The other parts were sound."

This case shews us how difficult it is to form a diagnosis between sanguineous and serous apoplexy! The septuagenary age of the woman—the pallid countenance—and the perfectly natural state of the respiration, were little indicative of both ventricles being filled with blood; and even in these *phlebotomising* days, would pretty certainly doom the man to an imputation of madness who took the lancet in his hand on such an occasion.

Yet does the acute Morgagni sanction venesection in these cases, and asserts that he saved the life of a relation of his, a nun, of 80 years, more than once, by bleeding and other evacuations, when she was attacked with apoplexy, regardless of the age, and only looking to the symptoms.

We shall introduce but one more case of apoplexy.

"Anthony Tita, author of the '*Catalogus Plantarum*,' died suddenly at Padua, in May 1729. The weather was then very hot, after long-continued rains and cold; and numbers of people were swept away with only a few hours' notice. Tita was 73 years of age, still robust and brawny; square figure, and somewhat fat. He was in the habit of exposing himself much to the sun; and to drink pretty freely, though not to drunkenness, of undiluted wines. He had been troubled, for some years, with ophthalmic inflammation, and lately complained of *fulness in his head.* On the 4th of May, the sun being unusually hot, having spent the whole day in the open air, and supped in the evening as usual, he suddenly cried out that he was very ill, lost the use of his tongue, and became hemiplegic on the left side. Morgagni immediately visited him. His senses were perfect; colour, respiration, and temperature, natural; pulse full and strong; complained of no pain, but seemed drowsy. Venesection from the *right* arm was performed; a smart purging glyster administered, and ol. succini applied to the olfactories. Morgagni having retired, one of Tita's own physicians came in, and thought proper to give the patient an emetic; soon after the operation of which, a still more violent attack was experienced, with stertor and convulsions, which ended next morning in death.

"*Sectio Cadaveris.* The dura mater adhered firmly to the skull. Its vessels (excepting the sup. long. sinus) were black from turgidity. Vessels of pia mater turgid also. *Right* lateral ventricle distended with blood of a black, coagulated appearance, and sufficient to fill the shell of a hen's egg. In the three other ventricles, there was fluid blood mixed with serum. The brain was sound and the substance of the hemispheres entire, so that it did not appear from whence the blood and serum could have issued. Towards the posterior part of each lateral ventricle, especially of the right, the plexus choroides had vesicles, the size of large grapes, full of water."

The propriety of exhibiting emetics in sanguineous apoplexy has occasioned much discussion, and even bitter and disgraceful litigation. That the *straining* during vomiting drives the blood with increased impetus through the arterial and capillary system to all parts of the body, and the head in particular, is most indubitable. This is evinced by the redness and swelling of the face, and accelerated velocity with which blood will flow from an orifice in a vein during the action of vomiting, as we have lately seen in some remarkable instances. It is stated, however, as the opinion of Dr. Parr, in the Lond. Med. Dict. that it is doubtful "whether, in every hæmorrhage, vomiting is not as useful, *by deriving to the surface*, as injurious from any other effect." We would just observe on this passage, that, in deriving blood to the surface from the interior, it *must* be driven through the capillary system into the veins, and consequently its course must be accelerated through the bleeding vessels, *wherever they are situated.* Surely then, to unload the vascular system itself, and particularly the vessels of the brain, by bloodletting, while determinations of blood to all, or any other parts, are solicited by those means which do not quicken, or much disturb, the general circulation, as purging, blistering, sinapisms, &c. are preferable to the hazardous experiment of vomiting, and its usual concomitant, straining. How often do we see apoplexy brought on by stooping to buckle the shoe, lifting a heavy weight, straining to evacuate a costive stool, &c.? in all which the blood is driven violently to the head through the arteries, while its return through the veins from thence is greatly impeded.

It could not escape the experienced eye of Morgagni, that apoplexy might occur from *turgescence* alone of the cerebral vessels, without any rupture of their coats, or exhalation of blood from their capillary terminations. Accordingly, he has related many examples of this kind of apoplexy, one of which we quoted in the first number of this series, page 9. These kinds of apoplexy, indeed, are the most dangerous and the most suddenly fatal of all, since the compression on the sensorium is universal. It appears to us that, in such cases, rupture or effusion (whether sanguineous or serous) procrastinates, and often saves the life of the patient. They are but partial evils, and to a certain extent, and in certain portions of the brain, are compatible with life; but general compression, to a certain degree, on the cerebrum and cerebellum, extinguishes the vital functions very quickly.

We may probably, in this way, account for the number of sudden deaths, in the night, which we continually hear of. A *gourmand*, whose vascular system is highly plethoric from immoderate eating and little exercise, goes to bed after a *hearty supper*, and falls asleep. The loaded stomach now presses on the descending aorta, and a disproportion of blood is thrown through the carotids and vertebrals to the brain. If the vessels give way, and either blood or water be poured out, apoplexy takes place, and the family are roused by the struggles or stertor of the patient. But if the vessels are too firm, then a general compression on the *whole* sensorium extinguishes the vital spark in a few minutes, and the man, who went to sleep in perfect health, is found, by his astonished friends, a cold and lifeless corpse in the morning!

At page 51 of Mr. Cooke's Abridgement, Mr. C. observes, that his own experience does not confirm the opinion of a relationship between hypertrophy of the heart and apoplexy. That relationship, however, has now been proved by so many pathologists, that the opinion and experience of an individual can have little weight in the other scale. Our own observations corroborate the more general opinion, and the three last and consecutive dissections which we have made, or been present at, of apoplexy, all displayed active dilatation of the left ventricle of the heart. Mr. Cooke subsequently observes thus: "In common with others, I have found the heart enlarged; and, whenever it acts with impetuosity, as is not unfrequently the case under these circumstances, it cannot be questioned, that the hazard of an apoplectic attack will be proportionately increased." So far, we think, Mr. Cooke's experience *does* corroborate the relationship abovementioned. We extract the following note of the translator, as containing judicious observations.

"Whatever tends to excite unnatural impetus of circulation; or to occasion undue determination of blood to the head, as appears to happen from protracted mental exercises, and from other affections of the nervous system, will have a tendency to induce a state of cerebral turgescence. Although not an advocate of the *exclusive*

agency of the digestive organs in the production of disease, I believe that derangement in their functions will often occasion the state of the nervous system here alluded to. At first perhaps the affection of the head is scarcely perceptible—the mind is dejected—the temper irascible—the head at length begins to ache, and the arteries to throb—an unexpected though slight concussion of the body seems to shake and distress the encephalon—there is frequent vertigo—and unless the disease be counteracted, apoplexy will not be an improbable consequence. I have known several cases of this nature, which bleeding, though temporarily mitigating the disorder, and removing from time to time the imminently threatening symptoms, did not cure, or even permanently relieve. Occasional blood-letting will perhaps be necessary, but it must be combined with assiduous attention to the secretions and excretions of the digestive organs, and to the quality and quantity of the ingesta." 52.

Although Morgagni does not rank himself among those who, "when they find a little water within the skull of an apoplectic person, immediately conclude that this was the cause of the disorder," yet he believes, there is sufficient reason for the division of apoplexies into sanguineous and serous. He seems to think, that the effused fluid is sometimes the *cause* of apoplexy by its pressure, and, sometimes, only the *effect* of that turgescence of the vessels, which, of itself, can induce the disease. This we imagine is good pathology.

The fifth Epistle of Morgagni is on Apoplexies which are neither Sanguineous nor Serous. An accurate examination of these cases will convince any one, that there was *pressure* on the cerebral or cerebellic mass, in every instance. We need only advert, in proof of this, to the case of the poor woman whose leg was amputated by Valsalva, and who did tolerably well till the third month, when, on the cicatrix healing, the patient was seized with a kind of apoplexy—"affectione corripitur de genere apoplecticarum," accompanied by delirium, convulsions, and diminished sensibility in the right side of the body, which carried her off in a few days. The head being opened, a large quantity of *pus* was found stagnating in the left ventricle of the brain, but no trace of injury or disorganization could be discovered in any part of the brain itself. It is hardly necessary to observe, that in whatever way this purulent collection was formed, it must have occasioned *pressure* on the brain, an occurrence which we believe to take place *invariably* in apoplexy.

Morgagni adverts to a circumstance in this Epistle, which has been more noticed in modern times than it then was. This is the fact, that there may be great determination of blood to the head, and great turgescence of vessels there, when patients have died, or appeared to die, of hæmorrhage.

Thus, "in a man," says Morgagni, "whose death happened from the rupture of a popliteal aneurism and the consequent effusion of blood, numerous bloody points appeared in the sections of the medullary substance, and were enlarged to drops when I made lateral pressure on the sections. The corpora striata of this patient, when cut into small pieces, exhibited no striæ, but a continued medullary band." The translator has appended a note to this observation, part of which we shall insert here.

"After uterine hæmorrhage, and also after copious depletion on account of pulmonary and other inflammations, I have frequently observed the symptoms of cerebral congestion—and which has generally appeared to arise from the excitement occasioned by some mental effort, though occasionally it has arisen without an evident cause. Whilst the other parts of the body appear comparatively bloodless, the vessels of the head throb violently; there is severe pain; confusion of intellect, sometimes to such a degree as to threaten delirium; the pulse at the wrist is usually small and vibrating, and the countenance distressed. When I first observed these symptoms I was led to abstract blood, from an apprehension of phrenitis; but I did harm: for if the urgency of symptoms was diminished, the susceptibility to a recurrence was increased, and restoration to health was protracted. The liability to this form of cerebral plethora has appeared to me to be proportionate to the preceding hæmorrhage, and the consequent debility. If in this condition an intrusive visiter be admitted to converse, though but for a short time, with the patient—or if the patient attempt to read, or in any other way to employ the mental faculties beyond what is perfectly easy—or if the mind be agitated, this state of the head will almost inevitably be induced. It may, however, be brought on by all those causes which tend to destroy the equilibrium of circulation; and none are more likely, in this condition of the patient, than noise in the room, deficiency of sleep, improper food, a constipated state of bowels, or a morbid state of the secretion into them."* 74.

The sixth section of Mr. Cooke's translation is on Paralysis—a disease so closely allied to apoplexy, as to be scarcely separable. On the subject of paralysis we have amply dilated in this Journal, when reviewing Dr. Cooke's able researches, and when analysing the writings of Rochoux, Bricheteau, Esquirol, and several distinguished continental papathologists. We shall, therefore, pass over this section, as

* In the first volume of the Medico-Chirurgical Journal and Review, (February 1816,) Dr. Seeds has detailed a suite of interesting experiments, on the comparative effects of venesection and arteriotomy. When death was caused by opening the *veins* of animals, a state of venous congestion was always found in the brain.

containing nothing with which our readers are not very familiar already.

The seventh section is on Epilepsy; and this subject also, has been fully treated of in some late numbers of our Journal. Morgagni almost invariably found organic disease of the brain in the dissection of epileptic patients. He sometimes, however, traces the origin of the disease to lesions of remote parts—these lesions producing, at the time of the epileptic paroxysm, a determination of blood to the head—and, if continued long enough, disease of structure there. We shall extract the 12th case, as it is rather remarkable, in the words of Mr. Cooke.

"Anastasia Poggi, a grave and virtuous priest, was seized with epilepsy in his sixty-eighth year. He was rather corpulent, and of a florid complexion. The first attack was preceded by pain in the right hypochondrium, and was removed by bilious dejections. The subsequent accessions were generally preceded by a sensation compared to the ascent of vapour from the hypochondria to the head. He was constantly annoyed with a sense of fulness about these parts, which was increased by taking food, but more especially after liquids. The pulse was slow. There was no symptom of affection in the head, till the disease had continued some considerable time, when his head felt heavy, and he had some dulness of intellect. Under these circumstances he repeatedly derived advantage from a diminution of the vascular plenitude by bleeding. The attacks were generally of short duration, but not slight in degree. The eyes were distorted, the limbs agitated, and all sensibility was suspended. There was frequently a sense of suffocation; and sometimes these symptoms were accompanied with stertorous respiration, and an involuntary flow of urine. When the quantity of urine was augmented, either spontaneously or under the employment of diuretics, the epileptic symptoms were frequently exasperated; but they were never mitigated by this occurrence. After various other means usually resorted to had proved unavailing, half a grain of opium, taken at the beginning of the night, rendered him essential benefit. By this treatment tranquil nights and comfortable sleep were obtained, though, previous to this, he was sometimes roused by sudden difficulty of breathing, which created an apprehension of hydrothorax. So far from the head being oppressed by the opiate, the heaviness and dulness which followed the daily attacks were removed; but when the opiate was omitted these symptoms returned. Having, on one occasion, passed an unusually disturbed night from that cause, the pulse became unequal; and other remedies having failed to adjust the deranged functions, the narcotic was again resorted to, by which quiet nights were secured; and not only was the inequality of the pulse decreased, but by persevering in the employment of it every night, the pulse, which had previously been slow, acquired a more natural degree of frequency.

"No fit having returned for thirteen days, the opium was omitted; but the patient again passed nights of restlessness and watching, and the attacks of difficult respiration became exceedingly troublesome. These affections too were allayed by a recurrence to the same medicine: the fits were reduced to one a month; and at length two months having elapsed without a single attack, I took leave of my patient.

"During the latter period of my attendance on this case, the opiate was only given occasionally. Throughout the treatment great attention was directed to the state of the chylopoietic viscera; for I believe these sudden incursions of disease originated from morbid actions in them, and not from redundant serum in the encephalon." P. 111.

Our readers are aware, that M. Esquirol has found affections of the spinal marrow in a considerable proportion of epileptics, on *post mortem* examination. Thus out of ten epileptic patients, who died at the Salpetriere between the 1st of February and the 1st of June 1817, nine were examined, and in seven of these, there were found lesions of the spinal marrow or its membranes. When we consider, that it is principally the voluntary muscles that are convulsed in this disease, and that this class is supplied by the spinal nerves, it is not unreasonable to suppose, that the vertebral brain is more frequently affected than was formerly believed, when the spinal column was so seldom examined.*

The eighth section is on spasmodic and convulsive diseases, and contains some interesting cases. The belief, founded on dissection, that irritation, or turgescence of vessels on the origins of nerves, is the great efficient cause of spasmodic diseases, now appears to gain ground. The eighth case in the section before us shews, that Morgagni's penetration did not allow him to pass this subject unobserved.

*Case.* "A wool-comber, twenty-one years of age, was attacked with fever, in the course of which he had an accession of delirium. Having experienced a remission of the mental wandering, he was brought into this hospital, where convulsive actions were immediately observed in the muscles of the upper limbs, and consequently subsultus tendinum in both wrists. There was no inflammatory crust on the blood which was withdrawn, but it was of an extremely dense and compact quality. An oppressive state of coma ensued; and three days afterwards he died." 123.

On dissection, having disunited the fifth and sixth vertebræ of the thorax, much serous fluid escaped from the spinal canal. The vessels of the pia mater on the posterior part of

---

* See Dr. Sanders' paper in the 5th vol. of the Medico-Chirurgical Journal and Review, p. 7, *et seq.*

the left hemisphere of the brain, were greatly distended with black blood. "The medulla spinalis being attentively examined from the cranium to the fifth dorsal vertebra, the vessels of its pia mater were so exceedingly turgid with blood, especially its posterior surface, as to resemble an injected part. Even the minute vessels of the spinal nerves participated in this turgescence."

We shall here extract a passage from an interesting paper published some years ago, by Dr. Moulson, Physician to the Halifax Dispensary, which bears strongly on the point under consideration.

"Having frequently been baffled in my attempts to cure spasmodic and convulsive diseases by purgatives, and having lost one or two children, when treated according to the principles laid down by the most celebrated practitioners who have written upon the subject, I was determined, the first opportunity that offered, to investigate anatomically the nature of these diseases, in hopes that a more successful practice might be deduced from appearances on dissection. Soon after these failures, a case occurred in the practice of my friend, Dr. Sanders of Edinburgh, which being proof against any internal remedies, I carefully noted down the different muscles spasmodically affected, that I might see whether any difference existed between them and those that were free from spasm. Permission being obtained to examine the child's body, the contents of the abdomen and thorax were found perfectly free from disease, but upon carefully examining the brain and spinal marrow, there appeared sufficient evidence to account for the spasmodic contractions, the cause of the child's death. The nerves distributed to the muscles previously noted down (whether proceeding from the brain or spinal marrow) were found to have at their *origins*,* the blood-vessels preternaturally turgid with blood, whilst the blood-vessels ramifying upon the nerves distributed to the muscles free from disease were perfectly natural. These appearances being put to paper, and reasoned upon at leisure, an opinion was formed, that could a sufficient quantity of blood be abstracted from those parts labouring under this turgescence, the disease would be removed. After this dissection, a case of chorea occurred in a girl about 14 years of age, and the purgative plan was prosecuted to the fullest extent: instead of the symptoms being alleviated by this treatment, they were aggravated, insomuch that she was obliged to be held down in bed by her father to prevent her from injuring herself. In lieu of this treatment, leeches were applied to the spine, followed up by blisters and frictions along its whole course, and by these means she completely recovered in a very few days. This is only one of the many examples I could adduce, of cases, in

"* By the word origin is here meant, that part of the brain or spinal marrow whence a nerve is seen to emerge immediately upon removing its membranes."

which the purgative plan failed, and which were relieved by the treatment above mentioned. Having found that abstraction of blood from as near as possible to the origins of the nerves distributed to muscles spasmodically affected had the effect of alleviating the disease, and that a cure was completed by blistering and friction; a case occurred in which I was determined to employ them without the aid of medicine.—A boy, about eleven years of age, was playing with his school-fellows, when suddenly he fell upon the ground in convulsions. When I first saw him, he had been an hour convulsed; the pupils of his eyes were widely dilated, mouth firmly closed, and his hands clenched. I immediately ordered eight leeches to the nape of the neck, desired that as much blood should be obtained as was possible, by the assistance of cloths wrung out of warm water; and that afterwards a large blister should be applied to the part. At two o'clock, P. M. I saw him first, when the leeches were ordered; at six, P. M. the blister was put on; and at ten, P. M. he was free from convulsions, and spoke quite rationally to his parents. The next day he was walking about the house, when I ordered him a laxative; and the third day from his attack I saw him flying a kite in the street. To enumerate cases of this kind, I think unnecessary; but must observe, that in every case of convulsions that has terminated fatally, I have invariably found turgescence at the origins of the nerves distributed to those muscles that were affected."*

In the prosecution of some experiments, Dr. Moulson had an opportunity of examining a horse that had died of what is termed the gripes. He could discover no visible cause of death, either in the abdomen or thorax; but upon opening the spine, he was astonished to find the blood-vessels, all along the spinal marrow, and particularly, those distributed upon the origins of the nerves going to the intestines, *gorged* with blood. The vessels of the brain were turgid, but in an inferior degree.

We observe that, in most of Morgagni's cases of tetanus, even of the traumatic kind, there were appearances in the brain or spinal marrow, indicative of inflammation or congestion.

The tenth case in Mr. Cooke's Abridgement, is a remarkable one, and we apprehend that Morgagni has misjudged it, at least, in an etiological point of view. He denominates it, "convulsions from deposition of serum, and turgescence of vessels, *originating in terror.*" Before offering a comment, we shall state the case itself, somewhat farther abbreviated.

A nightman, 50 years of age, plethoric, but "so addicted to liquor that he was often inebriated," was occupied in emptying the unwholesome privies of the hospital, in the night.

* See Med.-Chir. Journal, for May 1817.

This man suddenly fancied he saw a ghost, clad in white—was seized with tremor—his mouth became distorted by spasm—and in this lamentable condition, he was conveyed to bed. He was bled that night, and, also, next morning, "after which, he had still farther remission of the irregular muscular actions, and the pulse expanded and became febrile." Blood was again copiously abstracted, but the febrile symptoms continued, and, occasionally, the whole body was disquieted with spasm. His expression, by signs, indicated that he underwent severe pain in the head. He died six or seven days from the date of the fright.

"*Dissection.* The fingers were extremely rigid, but the arms were flexible. The intestines were inflated with gas; and the liver and spleen were of a bluish colour. The liver, too, was moderately enlarged.

"The vessels of the pia mater, even the most minute, were as turgid with blood as if injection had been urged into them; and this vascular turgescence even pervaded the lining membrane of the ventricles, and the medullary substance itself. When the commencement of the spinal marrow was lightly compressed, blood oozed from it, a circumstance which is rarely seen. Both the lateral ventricles abounded with a limpid fluid, and the choroid plexuses were florid. The fornix and the crura of the medulla oblongata were of a soft texture." 127.

Now, contrary to the opinion of Morgagni, we have no doubt, that the apparition was the *consequence*, and not the *cause*, of the disease. The habits of inebriation disposed the brain to vascular turgescence, and the phantom in the privy was nothing but a symptom of that disorder, which ultimately put a period to life. We have seen many instances, where inflammation or congestion in the brain was only indicated, at first, by some slight mental aberration, or anomalous symptom, that apparently had no connexion with so serious a state of an important organ, but where the event proved that most dangerous mischief was going on at the time.

The ninth section of the work before us is on Insanity. But our pages have, of late, presented far more important information on this subject than can be expected in the work of Morgagni. Eight cases are related, and the general pathological features were, induration of the cerebral substance, and serous effusion in the ventricles, or under the membranes of the brain.

"The brain has been found indurated by other anatomists. In my dissections the pineal gland often appeared diseased; and in almost all the dissections I have made of persons whose intellectual faculties were deranged, the substance of the cerebrum and cerebellum was unnaturally firm. The membranes of the brain have been thickened, and the falciform process ossified, both in cases of insanity and idiotism. It has occurred to me frequently to find that there had been determination of blood to the head, and more frequently deposition of serum beneath the membranes or in the ventricles. The spleen was probably often affected, and the liver and pancreas have also been found the seat of disorganization." 136.

Morgagni, however, admits, that indurated states of the brain have been observed without any disordered state of mind, and that intellectual aberration has existed where no structural alteration could be detected in the organ of thought. We queried the propriety of appending notes to the translation of Morgagni. We think the very long one, giving, among other things, "an abstract of thirty-seven cases from Haslam's Observations on Melancholy and Madness, *a work which is well known*," is very superfluous. If Haslam's work is "well known," which we grant it is, why reiterate his observations here? Or why append the observations of a few individual writers on madness to a work of a general nature like this? If notes are at all proper, they should be such as give the *present state of our knowledge* on the subject under discussion in as small a compass as possible, and not the peculiar opinions of a few individuals, still less insulated cases. We think Mr. Cooke, on mature deliberation, will see the propriety of these remarks. As we said before, the notes in themselves are extremely judicious and sensible—we only question the propriety of swelling out a work, already too large for modern readers, by any thing not in the original.

The tenth section is on hydrophobia: but Morgagni never had an opportunity of examining a hydrophobic body. Two or three dissections from his friends are given, but do not contain any thing satisfactory. The note which Mr. Cooke has appended to this section is so nearly what we conceive to be the proper kind of note (if notes at all are added) that we shall here extract it as a specimen of Mr. Cooke's manner and matter.

"Since the time of Morgagni the bodies of numerous hydrophobic persons have undergone minute investigation; but unhappily the result has not yet been such as to afford any greater confidence of mitigatiag the heart-rending symptoms which distinguish this disease, or such as to excite any expectation of averting its speedily fatal termination. This, perhaps, is the most justly dreaded of any malady to which the human body is exposed; and no practitioner merits the satisfaction of a peaceful mind, after the unsuccesful discharge of his professional duty, if he voluntarily resort to temporizing and useless expedients when he might extirpate the bitten part.

"It will not be compatible with my present undertaking to extend my remarks further than pointing out the general features of the cases alluded to; and in doing this I shall pass over the symptoms. Unfortunately they have been so often and so touchingly delineated, that to recapitulate them would be a work of supererogation.

"The time at which the symptoms of canine madness occur is extremely uncertain. There are insulated but strongly characterized instances in which their onset was observed within fourteen days of the bite, but those who have most frequently witnessed the disease, place their occurrence at a more distant period. The disease appears seldom to arise earlier than three weeks, and in most cases the intervening time exceeds this period, and extends to an indefinite term of weeks, months, or years. It will, however, be found to have transpired most frequently before two or three months have elapsed; but as far as we can rely on phenomena which pass under observation, and which associate the hydrophobic symptoms with a previous and suspicious bite, some years have glided away between the insertion of the poison, and the consequent disease. Morgagni alludes to the term of twenty and even forty years, but these statements must be received with great distrust.

"Occasionally this affection discovers itself before irritation completely ceases in the injured part, when as a precursor of the secondary disease the morbid action increases, and is propagated in the course of circulation, I may elucidate this by referring to a case related by Mr. Webster. A man was bit in the hand July 21st. He never entirely lost the sensation of pain in the part. On the 16th of August the pain became more severe, gradually extended up the arm to the shoulder and breast, and on the 19th the arm was immoveable. He was first visited by Mr. W. on the following day. The pain was excruciating, small cicatrices of a red colour were observable on the hand; and the man was unquestionably labouring under hydrophobia. Though comparatively of rare occurrence, there have been instances in which after complete cicatrization, and the entire cessation of excitement, the scars have again become inflamed, and the inflammation has been accompanied with itching and pricking sensations. In the generality of cases the wound has completely healed, and has ceased to awaken the least attention; and though sometimes a degree of redness comes on, in most cases there is no such premonitory token, or it is so slight as to elude observation. To this point, however, great attention ought still to be directed, for were it possible to determine only the frequent occurrence of this intimation, some hope may be entertained, that by timely interposition the horrible distress, and the melancholy catastrophe which otherwise await the unfortunate individual may after all be averted.

"On examination after death, no uniformity has been observed in the morbid appearances. In most cases there are striking marks of cerebral congestion; the vessels are loaded—indeed sometimes gorged with blood. The tunica arachnoides has occasionally been inflamed and thickened, and a redundance of serum has been found pervading the surface of the brain and distending the ventricles; and bubbles of air have been found blended with it. The structure of the brain is often exceedingly firm. The mucous membrane of the larynx, trachea, and bronchia, as well as of the pharynx and œsophagus present more constant appearances of augmented vascularity. This appearance has varied in degree; sometimes representing a slight blush of inflammation, at others the inflammatory action has been more conspicuous, but with equal if not greater frequency it has borne the aspect of congestion of blood, appearing, from the lividness of colour, as if the affected parts were verging to gangrene. The œsophagus has been found in a contracted state, and this tube, as well as the trachea, has been observed to be destitute of its natural moisture. An unusual prominence has been noticed in the papillæ of the tongue. The lungs are often the seat of excessive congestion of blood, and the pleura is occasionally inflamed. The heart is sometimes enlarged, and its vessels appear in a state of turgidity. The inner coat of the stomach frequently exhibits a plethoric condition of vessels with numerous spots, which seem to be owing to extravasation of blood; the rugæ being large and prominent. In this state of increased vascularity the diaphragm has not unfrequently participated. The liver and other viscera have occasionally presented indications of similar disorder, which might indeed be expected under such exquisite nervous susceptibility, such mental perturbation, and such vascular excitement, as are associated in this disease. The inequality in the distribution of blood is often denoted by the comparative state of the larger vessels after death, some of them being loaded with blood when others are empty. It is a circumstance worthy of notice that in many of these bodies, putrescence occurs soon after death.

"The infection is usually imparted by the bite of a dog or cat; in India, the jackal is also an agent of its propagation. M. Breschet is reported to have communicated the disease to a dog by inoculating him in the neck with the frothy saliva of a man under hydrophobia. Morgagni believed that the disease had even arisen from the virus having only fallen on the human skin, but this is scarcely credible.

"Symptoms which greatly resemble those of rabies contagiosa have arisen from moral impressions. An anecdote is related by Morgagni which shows how powerfully the mind may be agitated, even where the intelligent and professional character of the individual would have forbidden the expectation of such an occurrence. Alberto Fabbri, who was the first physician in Bologna, a little before Morgagni's time, was seized and strongly held by one hand, by a patient labouring under hydrophobia, while he was feeling the pulse with the other. He became so extremely dejected, as scarcely to command his reason, and the idea of self-destruction often occurred to him. For seven days he had abstracted himself from society, when his attention being rivetted through perpetual gloom, he was wetted to the skin under a heavy shower previous to his being conscious of it. The place was solitary, and before he could obtain shelter his melancholy was washed away. It is probable his imagination was influenced by a reliance on the efficacy of a sudden and unexpected profusion of water in averting hydrophobia. Here certainly no hydrophobic symptoms had arisen, but in other cases, from equally unwarrantable grounds, they have been developed; and perhaps but for the propitious shower, a modification of them, at least, might have been the destiny of Fabbri."—*Ed.* P. 155.

The eleventh section on aphonia and paraphonia contains but three cases, two of them depending on cerebral lesion, the third sympathetic of derangement in the digestive organs —a fruitful source of local disease, and local irregularity of action. We shall give some account of this last case.

A nobleman, 60 years of age, subject to bilious affections, gout, and hæmorrhoids, but from which he had lately been free, was seized with loss of voice in the beginning of May, attended with some difficulty of breathing, and a sense of constriction about the larynx.

"The aphonia suddenly came on, and as abruptly ceased, without any excretion. The periods of attack and duration were variable. Its continuance did not exceed two minutes, and often it was even more transient. He had an attack almost constantly about one o'clock in the morning, and it sometimes occurrred in the day. Indeed whenever he drank wine, and sometimes when he gaped, sneezed, or coughed, he was invaded by this affection.

"When seized he could take nothing into his mouth, nor rest in one place, but was constrained to walk about. The tonsils were slightly inflamed, and there was a copious discharge of acid humour by spitting." 159.

Blood was sparingly abstracted, first from the arm, and afterwards from the anus—gentle purges were given, and afterwards diluents. In consequence of these means he passed several nights without his usual annoyance. He was then advised to take a short journey, and he recovered in six weeks or two months.

We shall pass over Morgagni's Observations on diseases of the eye and ear, because the disorders of these organs have been investigated by modern surgeons with far more minuteness than in the days of our illustrious author. The section on diseases of the nose need not detain us, as there is nothing interesting in it. We were a little surprized to find a case of *apoplexy* under this head. There was only mere suspicion that the turgescent state of the cerebral vessels was determined by sneezing; and had this been demonstrable, still the case should have come in in the sections on apoplexy, regardless of the etiology of the disease. As well might we range tetanus among affections of the fingers, because it sometimes succeeds injuries of these parts.

The 15th section on injuries of the head, with and without fracture of the cranium, contains many cases that may be advantageously consulted by the practical surgeon.

The second chapter is on diseases of the thorax; and it may readily be imagined that it contains numerous interesting cases of lesions in the pleura, lungs, and heart. We cannot do any thing like justice to this important division of the work.

The most frequent organic disease of the lungs occurring in Morgagni's dissections was consolidation, or hepatization, the consequence of inflammation. Effusion, suppuration, tuberculation, are, we apprehend, of more frequent occurrence than canification of the lungs.

At page 302, Mr. Cooke has appended a note, containing the particulars of an interesting case that fell under his own observation. We shall somewhat condense it in this place.

On the evening of the 6th of March, 1820, Mr. Bates was seized with a rigor, succeeded by intense heat. In the evening of the 7th our author saw him. His pulse was then extremely rapid and firm, his mind having been a little confused, but now tranquil. He complained of violent pain in the chest, and aching of the limbs; his countenance and general appearance bespeaking a most formidable attack of disease. He was bled copiously—purged—and was ordered saline aperients. 8th, was found much better; but his pulse was rather quick, and he had some erratic pains in the limbs and joints. No rigors; bowels open. He was desired to live low, and use aperients. In three days he was down stairs, walking about a little, and proposed to visit London on the succeeding day. This was a fatal lull before a storm.

"On the evening of the same day febrile symptoms recurred, accompanied with some wandering pains: he passed a distressing night, and early the following morning my attendance was requested. He complained of almost intolerable pain in the loins, extending thence to the thorax, and especially through the region of the diaphragm, but rather on the right side. His respiration appeared to be carried on without the action of this septum. He had not evacuated the bowels for twenty-four hours; consequently there was some abdominal intumescence, but no tenderness. He experienced a sense of violent constriction of the chest; the pulse was a hundred and twenty; and the tongue yellowish. I took from him twenty ounces of blood, and instituted, both in relation to diet and medicine, a strictly antiplogistic plan." 303.

Dr. Uwins now joined in attendance. They agreed to repeat the bleeding, and to persevere with purgations aided by enemata.

"The ensuing morning we were informed that the pain continued to abate, but the tongue was flocculent and very dark coloured: the abdomen was tumid, and communicated to the patient the sensation of extreme fulness. He could scarcely respire; his countenance was deeply distressed; the angles of the mouth were depressed; and the pulse was a hundred and twenty. None of the means resorted to having proved effectual to open the bowels, we agreed to administer elaterium. He took, in divided doses, three quarters of a grain of Dr. Clutterbuck's preparation of that medicine. A few hours subsequent to taking the first dose, some extremely dark and offensive stools were voided; and between this time and our visit the following day, the bowels had been evacuated copiously; the evacuations were dark and bilious, the tongue was brown, the pulse was very small and weak, the eyes were sunk, and the countenance evinced that peculiar expression of deep solicitude so characteristic of serious organic mischief. He had hitherto subsisted on the most innutritious fluids, and still only partook of liquid aliment.

"In the middle of the day the evacuations were rather bloody, and exhaled a cadaverous odour. Though he was evidently in an almost hopeless state, his mind, naturally energetic, was buoyed up from an impression that he had experienced essential benefit since the intestinal evacuations.

"On the fourth day after the relapse, (March 13,) he reported himself conscious of increased weakness, but he had less pain, and could make a deep inspiration, and fully expand the chest without the least impediment. All the uneasiness experienced in this effort arose from some tenderness of the abdomen. He believed himself to be perfectly relieved from the thoracic affection,—and anticipated early restoration to health." 304.

On the 14th it was evident that death was at hand. The pain resulting from the attack upon the heart increased progressively through the day, and in the evening was unbearable. He died at eight o'clock next morning. The *post mortem* appearances we shall give in our author's own words.

*Dissection.* The external aspect of all the abdominal viscera I found perfectly natural, but the intestines, especially the colon, were inflated with gas. The mucous coat of the stomach was extremely red and appeared as if blood-shot; and this appearance extended through the duodenum: it became more slight in the other small intestines, but was again considerable in the cæcum. The villi were exceedingly turgid with blood. In the colon there was a remarkable cohesiveness of the fæcal contents to the mucous surface, which I found to arise from their adhesion to that kind of extraneous membrane so often formed upon inflamed surfaces, and which pervaded nearly the whole of this intestine.

"On attempting to open the thorax I found the cartilages had ossified, and in some places the intervening ligaments were almost in the same state—circumstances unusual at so early a period of life. As soon as the thorax was penetrated by dividing the fourth rib, serum and lymph began to issue; and when the sternum was reflected, I found the pleura pulmonalis on the right side strongly adherent, in several parts, to the costal pleura, and to the diaphragm. At the upper part of the chest, between the adhesions, about a pint and half of serum containing numerous flakes of coagulable lymph, was deposited; and below that fluid, nearly an equal quantity of pus, or, at least, a puriform fluid, was accumulated. This fluid was separated from the serous by parietes of coagulable lymph in the form of a dense but ragged cyst, resting upon the diaphragm, the pulmonary surface of which was highly inflamed throughout.

"The pericardium was occupied by about a pint of turbid fluid, like whey; the superficies of the heart was coated with lymph; and the inner surface of the pericardium was highly inflamed and likewise covered with this kind of concretion. The heart itself was small and empty; and the parenchymatous tissue of the lungs did not appear to be involved in the disease." 306.

Mr. Cooke has prefaced the article phthisis with a neat sketch of the opinions of Bayle, Portal, Baillie, Baron, Broussais, &c. We were not a little surprized to see that he took no notice of Laennec, who investigated the subject of tubercular phthisis with far more success than any of the authors mentioned.

The morbid anatomy of the heart and large blood-vessels occupies full one hundred and fifty pages, but we have not seen any thing on which we could dwell—the diseases of this organ being now far better understood than in the days of Morgagni. We observe, with surprize, that in the notes to this section also, Mr. Cooke quotes Corvisart and most of the writers on diseases of the heart, but never alludes to Laennec. This is quite inexplicable. We quote the following case from Mr. Cooke—a case which he considers as owing to "nervous irritability," but which we suspect will turn out, in the end, to be organic disease of the heart, however promising the appearances may be at the present time.

"Early in the morning of the twenty-fourth of last October I was requested to see a gentleman, about fifty years of age, who was labouring under extreme distress from pain in the region of the heart. The history given me was, that, for some months, the patient had been annoyed with flatulence and acidity in the stomach, and occasional dejection of mind; and within the last fortnight, especially after a little exertion, he had experienced oppression at the chest, and pain in the heart; but these attacks generally soon passed off. The paroxym, however, had continued throughout the night preceding my visit. The pain was diffused through nearly the whole of the left region of the chest, it extended to the shoulder, and down the arm to the ends of the fingers. He could make a deep inspiration without any material increase of suffering. His countenance did not evince a degree of disease equivalent to the distress he appeared to suffer. He said the agony was inconceivably violent, and peculiar in its nature; but though it had been constant during the night, the degree was occasionally heightened; and whilst these accessions lasted he felt as if instant death were inevitable. The left arm had been almost cold, but when I saw him it was nearly as warm as the right. The pulse, in this wrist, was scarcely perceptible, and occasionally it was intermittent; but the action of the opposite radial artery was strong, rather full, regular, and about a hundred and thirty in a minute. He had no pain in the head, nor any tendency to syncope. I regarded it, at this time, as a case of angina pectoris.

"About eight ounces of blood were withdrawn, and he took some hydr. submurias, with sulphate and carbonate of magnesia; and adopted an abstemious plan of diet. In the course of the day he had one or two severe attacks, but in the evening his circumstances were decidedly improved. On the twenty-fifth the pulsations were more equal; he experienced but little pain, though he distinguished a sense of weight in the region of the stomach, and some fulness in the fauces. On the twenty-sixth the pulsations were equal, about a hundred in the minute, and moderately soft. He now took an alkaline bitter, and an occasional dose of mercury, and recovered in a few days.—*Ed.*" 506.

Morgagni quotes a curious case from Ramazini of a young man in whom, for the space of four days, no pulsation could be felt in any of the arteries; yet he was strong and active, "and even the day of his decease he rose from bed and dressed himself. "During these four days he was perfectly cold, and did not micturate." No dissection is given. The editor of this Journal, in consultation with his friend Dr. Lara of Portsea, saw a very curious case of this kind, a few years ago, and as they had an opportunity of opening the patient after death, it may not be uninteresting in this place, though some particulars of the case were published in one of the early numbers of the monthly form of this Journal.

"*Case.* An old superannuated soldier, 65 years of age, corpulent, dropsical, and afflicted with ulcerated legs, applied for gratuitous relief on the 10th of January, 1817. There was evident fluctuation in the abdomen; the urine was scanty, and the legs œdematous. There was a peculiarity in the countenance, which induced the reporters to examine the region of the heart. No motion of that organ was perceptible in any position. The radial arteries were then felt for; but no pulse could be distinguished by either of the medical gentlemen present. The temporal artery on the left side was seen large and tortuous; but, on compressing it, no pulsation whatever was perceptible by either of the medical gentlemen. The other temporal artery also was destitute of pulsation. The carotids gave an obscure sensation of pulse. On being questioned if he felt different from what he had done for some days past, he replied in the negative. His health was bad, but not worse than usual. One of the medical gentlemen returned early the next morning to examine the patient, but found him in precisely the same state as to the arterial system. Some blue pill, aloes, squill, and digitalis, had been prescribed the preceding day. On the third morning one of the medical gentlemen returned. The patient said he had made a good deal of water, that his bowels were open, and that he felt much better. The pulsations were now perceptible in both radials and temporals. In the course of a week, the medicine seemed to lose its effect, and the urine to become again scanty. There was, however, an oozing of at least a pint of water daily from the ulcerated and œdematous legs. There was now evidently a great collection of water in the abdomen, and the patient informed Drs. Lara and Johnson, that he had once or twice lately felt some strange sensations in his chest, as though he were going to die. Great palpitation and anxiety in the region of the heart were felt at these times. On Wednesday, the 15th of January, the above-mentioned gentlemen visited the patient. They found him copying music, he having formerly belonged to the marine band. He said he found himself but indifferent. His urine was scanty, the abdomen much swelled, and still that indescribable expression of countenance which first attracted so much attention. All the arteries, however, were in full play, and distinctly pulsating. On the evening of this day, the nurse observed that the ulcers of the legs were suddenly dried up. He went to bed, at the usual honr, without complaining of any thing in particular, but was found dead next morning.

"The singularity of this case, in respect to non-pulsation of the arteries for such a long space of time, excited much curiosity as to the state of the heart; and with some difficulty, permission was procured to examine the thorax..

"*Sectio Cadaveris, thirty hours after death.* The face and neck quite livid with extravasated blood. The abdomen was an immense size with water, and anasarca prevailed pretty generally. The moment that the knife was pushed through the cartilages of the ribs, bloody serum gushed out in great quantities, and the lungs were protruded through the incision of the left side with much force. This was occasioned by the pressure of the diaphragm upon the thoracic viscera: it seemed indeed ready to give way every minute, and deluge us with the abdominal collection of water. After sponging out a considerable quantity of water, the lung of the left side was found healthy; that of the left, hepatized, and also anasarcous: it was firmly adherent to the pleura costalis in all places. The pericardium was tense, and contained a few ounces of serum. The heart was considerably and equally enlarged; at least one-third more than its natural volume. It appeared to be rather active than passive enlargement. The auriculo-ventricular opening of the left heart was also very large, but the margins of the valves presented cartilage and incipient ossification. The aorta was enlarged, but the semilunar valves were sound. The origins of the two coronary arteries were larger than the reporters ever remember to have seen; they would nearly admit the tip of the little finger. These arteries were slit open, and some of their branches appeared as large as the coronary arteries usually are found.

"It is not improbable that the liver was diseased; but the ascites precluded the idea of examination.

"The great peculiarity in this case is the remarkable phænomenon of non-pulsation of any of the tangible arteries, except the carotid, for so long a space of time, apparently without much inconvenience. The temporal artery was turgid and very distinctly visible. If any pulsation existed, it must have been easily felt. Are we to suppose, that there was no *motion* of the blood at this time through the vessels? or is it not more probable that the motion was so *languid*, in consequence of the embarrassed situation of the heart, that the phænomenon of pulsation was imperceptible, as in the veins, where the current is known to be so slow? When the bowels were set in motion, and the urinary secretion increased, the pulsations returned, and the heart obtained a momentary freedom of action. The drain from the legs procrastinated the fatal blow; and thoracic effusion, in its usual sudden and silent manner, snapped the thread of life without a moment's notice."

These cessations of pulsation in arteries we look upon as very suspicious symptoms in most cases. It is well known that this was one of the first things that attracted the notice of John Hunter to a disease that ultimately destroyed him. Feeling pain one day in the epigastric region, he accidentally observed, by looking in a glass, that his face was pallid as a corpse; and on applying his fingers to the wrist, there was no pulsation in the arteries. His breathing could only be carried on by *voluntary* exertion. In this state he continued three quarters of an hour, feeling as if death would inevitably take place, if respiration was not carried on by voluntary efforts. He died in one of these paroxysms, nearly twenty years after the first accession of the disease, during a fit of passion in St. George's Hospital, in October 1793. On dissection, the coats of the stomach and intestines appeared loaded with blood. The pericardium was thickened, so that it would not collapse on being slit open. The heart was very small, and its muscular structure paler and of looser texture than natural. The coronary arteries were converted into bony tubes. The mitral valves were ossified in some places, and the aortic valves were rather indurated. There was also incipient aneurism of the aorta.

We shall here insert a case of disease of the heart, detailed by Morgagni in his 24th epistle, article 13.

A middle-aged man came into the hospital in the spring of 1705, complaining of pain in the right hypochondrium, where there was an evident enlargement, apparently of the liver. The pulse was remarkably small and frequent.

" About four hours after coming into the hospital, he was attacked with such violent pain in what he designated the stomach, that from its severity, and the paleness of his face—from the cold perspiration which attended it—from the pulse being almost imperceptible—and from the respiration being in the state it generally is in dying persons, he appeared actually on the point of death. From this attack, however, he recovered, and related to the persons around him, that he had several times undergone similar paroxysms. His pulse returned to the state I have described. On the following day the physician ordered blood to be withdrawn from the arm, and directed the administration of suitable medicines. The tumour of the liver gradually disappeared after a few days, when the man was seized with pain in the region of the heart, accompanied with difficulty of breathing. A small quantity of blood was again withdrawn, by which the respiration was somewhat relieved, but the state of pulse was only improved in a slight degree—indeed it was so small and languid, in the temples as well as at the wrists, as to be scarcely perceptible. I applied my hand to the left side of the chest, and found that the heart beat with equal frequency, and with moderate power. The action of this organ was sensibly felt much below the region it occupies. It seemed to labour, and the patient solicited what are commonly termed cordial medicines. About the eighth or ninth day after coming into the hospital he died suddenly.

" *Dissection.* The pericardium contained a large quantity of a yellowish serum; and, from enlargement, as well as the accumulation of fat on the heart, that organ was the most unsightly I had witnessed. The small vessels of the lungs were black, and distended with blood; the texture of the lungs, between the vessels, was whitish, except at the upper part, where, both externally and internally, the tissue was black and extremely indurated; and when cut into a thickish fluid of a tobacco colour issued from it.

The liver was indurated and marbled; and not only exhibited small white spots, but some also which were of the colour just mentioned. The coats of the gall-bladder were black, and that receptacle contained bile which in colour resembled ink, although the contiguous pylorus and duodenum were tinged yellow." 511.

We not unfrequently meet with cases in which the disordered action of the heart is such as to lead to a conviction of organic disease in that viscus, and yet the event fortunately belies the unfavourable conclusion drawn. There is a remarkable instance of this kind related by Dr. Clutterbuck in the first volume of the Transactions of the Medical Society of London, where a female recovered by rest and judicious evacuations from a state apparently hopeless. We may be permitted to give a greater publicity to this case than it has ever yet received, by stating some of the particulars in this place.

Mrs. C. ætat. 35, married, applied to Dr. C. October 29th, 1814. General appearance exceedingly distressing; countenance expressive of great anxiety; skin perfectly pallid and ex-sanguine, except lips and cheeks, which bore a leaden hue; tongue clean, moist; ex-sanguine; extremities cold; pulse weak and irregular; breathing much oppressed; face bloated; legs œdematous to the knees; constant uneasiness in the region of the heart; frequent palpitations, even when in bed; always upon walking; pulse at the wrist irregular during palpitation. Upon making any unusual exertion, the cardiac uneasiness is aggravated to the degree of acute pain, extending to the back, collar bones, and middle of the upper arms, particularly the left. Menses regular in period, but trifling in quantity, and nearly colourless. Appearance altogether chlorotic, with not a few symptoms of hydrothorax, or hydro-pericardium. Appetite very bad; great uneasiness after eating; constant constipation; general strength greatly reduced; apparently in a very dangerous state, of which she herself was sufficiently aware.

These symptoms had continued for several months, gradually increasing. They commenced soon after an inflammation in the chest.

These symptoms sufficiently indicated an excess of irritability and disordered action in the heart; while their duration, severity, and their having succeeded an attack of thoracic inflammation, gave reason to apprehend some disorganization in the structure of that viscus. Notwithstanding the debility and exsanguineous state of the patient, it was feared that inflammatory action was still going on, and therefore about five ounces of blood were abstracted from the arm, which the patient bore without inconvenience, and evident relief was obtained. Digitalis was administered in small and frequent doses, as was also ammonia, with the view of exciting a little action in the stomach, and of determining to the surface. Several evacuations were daily procured from the bowels by aperients; and plain easily-digested food was allowed, but all strong drinks prohibited. Perfect quietude and the horizontal position were enjoined—she was confined almost entirely to bed for ten weeks. The blood-letting was repeated at intervals, and the plan altogether persisted in for nearly three months, with gradual and continued amendment. At the end of that period her health was perfectly restored. Dr. Clutterbuck very justly remarks that, had this case been treated with tonics and stimulants (medicines that are too often had recourse too under similar circumstances) the disease would, in all probability, have been aggravated, and the patient's life been lost.

Dr. Bourne, of Coventry, has also detailed a very instructive case of this kind, in the 4th vol. of the Medico-Chirurgical Journal, where disordered action of the heart prevailed to a very great extent—where the pulse was extremely feeble, irregular, and intermittent, so that scarcely a distinct beat could be reckoned every three or four seconds, with fluttering undulatory vibrations intermediately. The surface of the body was pale and cool—the legs œdematous—urine scanty—and the patient could only lie on her back with her head raised. Under these circumstances a small quantity of blood was abstracted from a vein, which seemed to improve the state of the symptoms, while small doses of calomel, antimonial powder and digitalis were exhibited internally. By these, and a few other remedies of a similar tendency, the patient was saved for that time, and had apparently recovered, with the exception of some little irregularity of pulse which still continued. She pursued her domestic concerns till the ensuing spring, (nearly six months,) when the same symptoms returned, but she then put herself into the hands of a quack, who soon, as may be readily supposed, consigned her to the care of the undertaker. These cases are, in truth, exceedingly fallacious, in their appearances, and uncertain in their terminations. We lately witnessed a very remarkable instance of the kind. A young lady had laboured for more than two years with all the usual symptoms of active enlargement of the heart, attended with great sufferings, emaciation, œdematous swellings, and other formidable phænomena. When apparently at the brink of the grave, she took a turn for the better, and nearly lost the whole of the abovementioned symptoms. When appearances were thus so flattering, and the friends were beginning to taunt the doctor about his false prognosis, the train of morbid phenomena returned, and now put a period to her existence in the course of two or three months.

Morgagni makes many ingenious and just observations on the pulse. He mentions the case of an old man who had had epilepsy from an abdominal affection, and in whom he found the pulsations only 22 in the minute; which sluggishness had existed for several months, though the man was able to walk about like a healthy person. We have seen four or five instances where the pulse was at or under 30 in the minute; but in three of these cases the pulse, when any little occasional excitement took place in the system, rose to just double the number of the ordinary rhythm. From this we have been led to conclude that, under such circumstances, every second contraction of the left ventricle failed to produce the sensible phenomenon of the pulse, though the circulation went on nearly the same as in the ordinary state of the heart and arteries. The last case of this kind which came under our notice, was the late Mr. Busby of Bond Street, whom we attended in consultation with Mr. Cosgreave, an intelligent practitioner in Surry Street. His pulse had been for years at 28 in the minute; but on several occasions it rose all at once to 56 and even more. He was subject to a train of exceedingly anomalous symptoms, attended with long paroxysms of syncope. He died rather suddenly about twelve months ago, and we could not obtain permission to open the body. We have no doubt, however, that there was *valvular* disease of the heart. He was nearly seventy years of age.

We shall extract the following case from Morgagni, to shew that he has described the disease, since more fully delineated by Dr. Baron, under the appellation of "tuberculated accretions of the serous membranes."

"A little before the close of the year 1704, a lad experienced some difficulty of breathing, which arose without any evident cause. He was received into the hospital of St. Mary de Morte, and different methods of treatment were employed, but without success. He was repeatedly bled, and the dyspnœa was alleviated for a time by this means; but though purgative medicines did not appear to be injurious, he was not benefited by them in the slightest degree. These circumstances were related to me when I first saw the patient, which was scarcely three days before his death. He was then pallid, and the state of his respiration constantly required the erect position. During the act of inspiration I observed that the lower part of the chest was greatly elevated. He had no thirst, the temperature of the skin was not hot, nor had he any other indication of fever. The arterial pulsation was frequent, but, when the hand was applied to the chest, the palpitation of the heart appeared to be much more frequent than the pulsations of the arteries. This comparison was repeatedly and attentively instituted, and the contrariety I have mentioned was uniformly observed. The actions of the heart and arteries were astonishingly unequal. The difficulty of breathing having greatly increased, he died about the hundredth day from the commencement of the disease. At the time of his decease the face was swollen, but the feet were not œdematous.

*Dissection.* The face was still tumid, and there was a degree of lividness about the eyes and abdomen. The omentum appeared to be of a blackish colour, and both its surfaces were covered with globular bodies like glands. The liver was externally white, and internally it approached a tobacco colour. It had contracted unnatural adhesions to the adjacent parts, especially to the septum transversum. The peritoneum also, where it invests the diaphragm, was rugged from globular substances, which varied in their size and figure. The abdominal cavity contained a redundance of serous fluid of a yellowish green colour.

Both the thoracic cavities were filled with fluid like that effused into the abdomen, and flakes of lymph, resembling thin membranes, floated in it. The right lung adhered to the costal pleura, and the posterior part of this membrane presented an appearance like ecchymosis, and the blood extravasated here was of a crimson hue. The left lung, at its upper and lateral parts, was firmly annexed to the pleura, which in those places, and also the pleuritic covering of the subjacent diaphragm, and of the anterior part of the mediastinum, was not only beset with round bodies similar to those which pervaded the peritoneum, but the membrane had acquired a degree of hardness and thickness which exceeded the density of the coats of the aorta at its origin. The internal texture of the pleura consisted of a white substance, made up of minute particles. When the lungs were compressed they were observed to be full of frothy ichor. The pericardium scarcely contained more fluid than is usually found in that bag, but the fluid exhibited the same appearance with that which occupied the other cavities." 520.

Even the *hydatid* origin of these tubercles is alluded to by Morgagni, as the following passage will shew.

"It is difficult to determine the cause of the appearances which presented themselves in the pleura. Hippocrates and Galen entertained the opinion, that serous fluid may be accumulated in the thorax and pericardium from ruptured hydatids, which had been observed in the ox, the dog, and the sow; and from their existence in these animals it was inferred that the human body was liable to them, in a ratio of increase proportionate with its greater tendency to disease." 528.

We must now close our first article with the first volume, and shall, in our next number, present our readers with a series of cases and observations from the other volume. We have furnished sufficient specimens both of the translation and notes, to enable the reader to form a very correct opinion of both the one and the other.

